# PRAGMATIC, SUSTAINABLE, AND COST-FEASIBLE TECHNOLOGIES TO ENABLE REMOTE CARE COORDINATION SERVICES

**DOI:** 10.1093/geroni/igad104.0314

**Published:** 2023-12-21

**Authors:** Knoo Lee, Suzette Bacon, Elizabeth Conrow, Elizabeth Curtis, Ashley Roberts, Blaine Reeder

**Affiliations:** University of Missouri, Columbia, Missouri, United States; Sinclair School of Nursing, University of Missouri, Columbia, Missouri, United States; Sinclair School of Nursing, University of Missouri, Columbia, Missouri, United States; Sinclair School of Nursing, University of Missouri, Columbia, Missouri, United States; Sinclair School of Nursing, University of Missouri, Columbia, Missouri, United States; Sinclair School of Nursing, University of Missouri, Columbia, Missouri, United States

## Abstract

The aim of this presentation is to communicate the approach and lessons learned in successfully deploying open source smart home and consumer-grade technologies to enable novel remote care coordination services for older adults as part of the CMS-funded ASSETs for Aging in Place demonstration project. Major barriers to use of technologies that enable service reach to rural or home-bound at-risk populations are technology availability, internet connectivity, cost, configuration, installation, ease of management, and usability and usefulness of clinically relevant data displays for decision-making to support personalized care coordination, coaching, and self-management goals. We have pioneered a reusable, innovative approach and infrastructure through partnership with the State of Missouri Department of Social Services and a non-profit hosting partner that facilitates testing and deployment of such remote services with broad goals of scalability and translation as starting principles. The specific focus of this presentation will describe technology selection, configuration, proof-of-concept testing, use of mobile hot spots to overcome broadband availability in rural areas, team composition and skills, introduction and training of the OT/Nurse/Social Worker teams to technology, resources required to stand up the team and transition to independent technology expertise in the field with mobile, video, and remote messaging tools, and user-centered design engagement of the care coordination and informatics teams to drive dashboard features and visual display developments for new work flows. An overview of the approach, technologies, and practical considerations to support research and development of new technology-enabled services for older adults will be presented.

